# Improving social justice in observational studies: protocol for the development of a global and Indigenous STROBE-equity reporting guideline

**DOI:** 10.1186/s12939-023-01854-1

**Published:** 2023-03-30

**Authors:** Sarah Funnell, Janet Jull, Lawrence Mbuagbaw, Vivian Welch, Omar Dewidar, Xiaoqin Wang, Miranda Lesperance, Elizabeth Ghogomu, Anita Rizvi, Elie A. Akl, Marc T. Avey, Alba Antequera, Zulfiqar A. Bhutta, Catherine Chamberlain, Peter Craig, Luis Gabriel Cuervo, Alassane Dicko, Holly Ellingwood, Cindy Feng, Damian Francis, Regina Greer-Smith, Billie-Jo Hardy, Matire Harwood, Janet Hatcher-Roberts, Tanya Horsley, Clara Juando-Prats, Mwenya Kasonde, Michelle Kennedy, Tamara Kredo, Alison Krentel, Elizabeth Kristjansson, Laurenz Langer, Julian Little, Elizabeth Loder, Olivia Magwood, Michael Johnson Mahande, G. J. Melendez-Torres, Ainsley Moore, Loveline Lum Niba, Stuart G. Nicholls, Miriam Nguilefem Nkangu, Daeria O. Lawson, Ekwaro Obuku, Patrick Okwen, Tomas Pantoja, Jennifer Petkovic, Mark Petticrew, Kevin Pottie, Tamara Rader, Jacqueline Ramke, Alison Riddle, Larissa Shamseer, Melissa Sharp, Bev Shea, Peter Tanuseputro, Peter Tugwell, Janice Tufte, Erik Von Elm, Hugh Sharma Waddington, Harry Wang, Laura Weeks, George Wells, Howard White, Charles Shey Wiysonge, Luke Wolfenden, Taryn Young

**Affiliations:** 1 Department of Family Medicine, Queen’s University, Kingston, Canada; 2 Department of Family Medicine, Faculty of Medicine, University of Ottawa, Ottawa, Canada; 3 School of Rehabilitation Therapy, Faculty of Health Sciences, Queen’s University, Kingston, Canada; 4 Department of Health Research Methods, Evidence, and Impact, McMaster University, Hamilton, Canada; 5 Bruyère Research Institute, Bruyère Continuing Care and University of Ottawa, 85 Primrose, Ottawa, Ontario Canada; 6 School of Epidemiology and Public Health, University of Ottawa, Ottawa, Canada; 7 Michael G. DeGroote Institute for Pain Research and Care, McMaster University, Hamilton, Canada; 8 School of Psychology, University of Ottawa, Ottawa, Canada; 9 Department of Internal Medicine, American University of Beirut, Beirut, Lebanon; 10 Canadian Council on Animal Care, Ottawa, Canada; 11 International Health Department, ISGlobal, Hospital Clínic - Universitat de Barcelona, Barcelona, Spain; 12 Centre for Global Child Health, Hospital for Sick Children, Toronto, Canada; 13 Institute for Global Health & Development, The Aga Khan University, Karachi, Pakistan; 14 Indigenous Health Equity Unit, Melbourne School of Population and Global Health, The University of Melbourne, Melbourne, Australia; 15 Judith Lumley Centre, School of Nursing and Midwifery, La Trobe University, Melbourne, Australia; 16 MRC/CSO Social and Public Health Sciences Unit, School of Health and Wellbeing, University of Glasgow, Glasgow, UK; 17 Unit of Health Services and Access, Department of Health Systems and Services, Pan American Health Organization (PAHO/WHO), Washington, DC USA; 18 Doctoral School, Department of Paediatrics, Obstetrics & Gynaecology, and Preventive Medicine, Universitat Autònoma de Barcelona, Barcelona, Spain; 19 Malaria Research and Training Center, University of Sciences, Techniques, and Technologies of Bamako, Bamako, Mali; 20 Department of Psychology, Department of Law, Carleton University, Ottawa, ON Canada; 21 Department of Community Health and Epidemiology, Dalhousie University, Halifax, Canada; 22 School of Health and Human Performance, Georgia College, Milledgville, USA; 23 Healthcare Research Associates, LLC/S.T.A.R. Initiative, Los Angeles, USA; 24 Well Living House, Li Ka Shing Knowledge Institute, University of Toronto, Toronto, Ontario Canada; 25 Waakebiness Institute for Indigenous Health, Dalla Lana School of Public Health, University of Toronto, Toronto, Canada; 26 General Practice and Primary Healthcare, University of Auckland, Auckland, New Zealand; 27 WHO Collaborating Centre for Knowledge Translation and Health Technology Assessment in Health Equity, Ottawa, Canada; 28 Royal College of Physicians and Surgeons of Canada, Ottawa, Canada; 29 Applied Health Research Center, St. Michael’s Hospital, Toronto, Canada; 30 Dalla School of Public Health, University of Toronto, Toronto, Canada; 31 Liverpool School of Tropical Medicine, Liverpool, UK; 32 School of Medicine and Public Health, The University of Newcastle, Callaghan, New South Wales Australia; 33 Centre for Evidence Based Health Care, Department of Global Health, Stellenbosch University, Stellenbosch, South Africa; 34 Cochrane South Africa, South African Medical Research Council, Cape Town, South Africa; 35 Interdisciplinary School of Health Sciences, Faculty of Health Sciences, University of Ottawa, Ottawa, Canada; 36 Africa Centre for Evidence, University of Johannesburg, Johannesburg, South Africa; 37 The BMJ, London, UK; 38 Department of Epidemiology & Biostatistics, Institute of Public Health, Kilimanjaro Christian Medical College, Moshi, Tanzania; 39 University of Exeter College of Medicine and Health, Exeter, UK; 40 Department of Family Medicine, McMaster University, Hamilton, Ontario Canada; 41 Department of Public Health, Faculty of Health Sciences, The University of Bamenda, Bamenda, Cameroon; 42 Clinical Epidemiology Program, Ottawa Hospital Research Institute, Ottawa, Canada; 43 College of Health Sciences, Makerere University College of Health Sciences, Kampala, Uganda; 44 Department of Family Medicine, School of Medicine, Pontifica Universidad Católica de Chile, Santiago, Chile; 45 Faculty of Public Health and Policy, London School of Hygiene and Tropical Medicine, London, UK; 46 Department of Family Medicine, Epidemiology and Biostatistics, Western University, London, Ontario Canada; 47 Freelance Health Research Librarian, Ottawa, Canada; 48 International Centre for Eye Health, London School of Hygiene & Tropical Medicine, London, UK; 49 School of Optometry and Vision Science, University of Auckland, Auckland, New Zealand; 50 Health Research Board Centre for Primary Care Research, Department of General Practice, Royal College of Surgeons in Ireland, Dublin, Ireland; 51 Department of Medicine , University of Ottawa, Ottawa, Ontario Canada; 52 Hassanah Consulting, Seattle, WA 98122 USA; 53 Cochrane Switzerland, Centre for Primary Care and Public Health (Unisanté), University of Lausanne, Lausanne, Switzerland; 54 London International Development Centre, London School of Hygiene & Tropical Medicine, London, UK; 55 Canadian Agency for Drugs and Technologies in Health, Ottawa, Ontario Canada; 56 University of Ottawa Heart Institute, Ottawa, Canada; 57 Campbell Collaboration, Oslow, Norway; 58 HIV and other Infectious Diseases Research Unit, Durban, South Africa

**Keywords:** Reporting guidelines, Health equity, Social justice, Observational studies

## Abstract

**Background:**

Addressing persistent and pervasive health inequities is a global moral imperative, which has been highlighted and magnified by the societal and health impacts of the COVID-19 pandemic. Observational studies can aid our understanding of the impact of health and structural oppression based on the intersection of gender, race, ethnicity, age and other factors, as they frequently collect this data. However, the Strengthening the Reporting of Observational Studies in Epidemiology (STROBE) guideline, does not provide guidance related to reporting of health equity. The goal of this project is to develop a STROBE-Equity reporting guideline extension.

**Methods:**

We assembled a diverse team across multiple domains, including gender, age, ethnicity, Indigenous background, disciplines, geographies, lived experience of health inequity and decision-making organizations. Using an inclusive, integrated knowledge translation approach, we will implement a five-phase plan which will include: (1) assessing the reporting of health equity in published observational studies, (2) seeking wide international feedback on items to improve reporting of health equity, (3) establishing consensus amongst knowledge users and researchers, (4) evaluating in partnership with Indigenous contributors the relevance to Indigenous peoples who have globally experienced the oppressive legacy of colonization, and (5) widely disseminating and seeking endorsement from relevant knowledge users. We will seek input from external collaborators using social media, mailing lists and other communication channels.

**Discussion:**

Achieving global imperatives such as the Sustainable Development Goals (e.g., SDG 10 Reduced inequalities, SDG 3 Good health and wellbeing) requires advancing health equity in research. The implementation of the STROBE-Equity guidelines will enable a better awareness and understanding of health inequities through better reporting. We will broadly disseminate the reporting guideline with tools to enable adoption and use by journal editors, authors, and funding agencies, using diverse strategies tailored to specific audiences.

**Supplementary Information:**

The online version contains supplementary material available at 10.1186/s12939-023-01854-1.

## Background

The COVID-19 pandemic has magnified existing inequities in health and revealed systemic biases and discrimination in societal structures (such as racism, sexism, ableism), including health systems [1, 2]. Health inequities are defined as disparities that are avoidable and unfair [3, 4]. Acknowledging and addressing health inequities has been the subject of investigations and reports dating back to the 19^th^ century, with continued commissions including the Black report in 1980, WHO Commission on Social Determinants of Health in 2008 and National Academies of Sciences, Engineering and Medicine in 2017 [5–9].

Health inequities are driven by structural inequities in opportunities and resources, which shape the conditions in which people live and their opportunities for health [10]. By structural inequities we mean the unequal distribution of power, resources, and privilege that favour some people and place others in vulnerable or disadvantaged circumstances, limiting their health opportunities across gender, age, ethnicity, and other sociodemographic factors [4, 11, 12]. Such limitations lead the most vulnerable due to systemic and structural barriers to struggle to adequately meet their health care needs [13].

Many nations and peoples have suffered the deleterious effects of colonization or control by external military and political forces throughout history. This has resulted in many nations continuing to struggle with inadequate health and health care resources. For example, in India, healthcare workers are unable to maintain standards of care due to colonial-era regulatory policies creating disputes between federal and union government that impact resource allocation [14]. Moreover, wealthier countries continue to fail to honour global treaties to support health and health research, exemplified by profound inequities in access to vaccination for COVID-19 globally, with only 4% of people vaccinated in low-and-middle-income countries (LMIC) compared to over 70% in Canada and the United States of America (USA) [15, 16] in the year 2021.

In many countries, specific ethnic groups, such as Indigenous people, continue to suffer from the legacy of colonization and persistent discrimination and oppression [17–19]. Despite global instruments such as the United Nations Declaration on the Rights of Indigenous Peoples (UNDRIP) [20] to build a more just and equitable future, Indigenous people continue to face individual and systemic discrimination, limiting their opportunities for education, justice, and health [21]. Research about Indigenous people has often failed to partner with communities and consequently generated little social value for communities [22]. Instead, research has a legacy of exploitation and harm of Indigenous people through experimentation (e.g., the recently documented experiences of Indigenous peoples in Canada at Indian hospitals [23] and residential schools [24], Qu’Appelle BCG Vaccine Trial in Canada [25]. Historical exploitation has contributed to a culture of mistrust of health research in Indigenous populations, which must be considered in conducting Indigenous-centric health research. For Indigenous people, factors that drive opportunities for health may differ from other populations, such as the right to self-determination, and connection to the land and culture, which is central to wellbeing [26, 27].

Studies where the researcher is not in control of the exposure or intervention are known as observational studies. These studies could be prospective or retrospective or both. Observational studies have become increasingly popular as a key tool in national and international health monitoring, such as demographic health surveillance systems. Furthermore, there is an increasing focus on harnessing “Big Data” to link socioeconomic, administrative, and other biomedical data to inform planning [28–32]. Observational studies are also used to evaluate the effects of policies and programs in real-world situations, especially when an experimental study (e.g., randomized controlled trial) would be impractical or unethical [33]. Observational studies are also meaningful for exploring complex systems and their influence on health [34–37]. The informative value of results from interventions (e.g., policies and programs) addressing to mitigate inequities relies on the quality of data. However, the availability of data to monitor, understand and intervene to mitigate health inequities between and within countries continues to be a challenge [38, 39].

Implementing programs and policies to mitigate inequities effectively requires high quality data to monitor the impacts of policies and programs on health inequities over time and across settings. Many observational studies fail to assess inequitable differences in health or health care due to inadequate collection or reporting of equity-related data in participants, adjusting for available sociodemographic factors as confounders rather than exploring potential for differential effects for populations experiencing inequities, or due to a failure to evaluate variation in treatment effects [40–42]. Globally, decision-makers have endorsed the need for better evidence about health equity, exemplified by the Sustainable Development Goals (SDGs) of the UN General Assembly [4]. The Pan-American Health Organization (PAHO), the World Health Organization (WHO) and the World Health Assembly have all explicitly advocated for standards in reporting and analyzing equity [43–46]. Academic support for better evidence on health inequities is exemplified by an increasing number of global and national guidelines on assessing sex, gender and health equity, such as the 2015 Sex and Gender Equity in Research (SAGER) guidelines [47] and funder guidelines on considering sex, gender and health inequities [48, 49]. Justice and equity concerns are also central to research ethics [50, 51].

Reporting guidelines have been shown to improve the reporting of some aspects of their respective studies [52, 53]. The STROBE (STrengthening the Reporting of OBservational studies in Epidemiology) guidelines were developed to improve the reporting of observational studies, including cohort, case control and cross-sectional studies. Of the 13 STROBE extensions addressing nuances of design and types of data; none focus on health equity [54]. The development of equity extensions for STROBE items may improve the reporting of equity in observational studies.

Given the pervasiveness of observational studies and the growing attention to the need to examine inequities, especially inequities observed during the COVID-19 pandemic, methodological research, and guidance to improve their reporting is needed to improve the social value and global impact of health research.

### Overall goal

Our overall goal is to develop guidance on reporting health equity considerations in observational studies to make health equity data more readily available for synthesis and decision-making.

This goal has two distinct components, which are:

1. to develop a global reporting guideline on health equity as an extension of STROBE in partnership with members of the public, diverse collaborators, and multiple disciplines, and
2. to develop a reporting guideline for reporting health equity in Indigenous research in Australia, Canada, New Zealand and the USA with leadership by Indigenous people from these countries.

We recognize that the harms of colonization have affected Indigenous Peoples globally. However, we decided to focus on these four countries since they have a similar experience of colonization which included land dispossession and policies for assimilation [55]. We sought Indigenous scholars from each of these countries to join our Indigenous Steering Committee and designed methods to collect data on research methods in these countries (see below). We considered that this project did not have sufficient resources to adequately appraise the empirical data and seek sufficient global representation to develop reporting guidelines for all global Indigenous Peoples. By transparently reporting our methods and processes, we hope this guideline may be helpful for adaptation for other Indigenous Peoples in different global contexts. Indigenous Peoples, scholars, public members will be given opportunities to review the global consensus guidelines as well. We feel the entire project benefits from Indigenous perspectives.

The research to achieve this goal will be interconnected and empowering, with opportunities for reciprocal learning through joint meetings and communication. Indigenous approaches to research include taking a strengths-based, anti-oppressive and anti-colonial approach [56], founded on authentic, ethical partnerships [57, 58] and following principles of Indigenous data sovereignty [59]. These approaches are central to understanding health inequities and social justice in designing, conducting, and reporting research.

### Specific objectives

This project aims to improve the consistency of reporting of health equity in observational studies, by fulfilling the following specific objectives over the span of 4 years:

1. To assess reporting of health equity through empirical studies by:
  1.1. identifying available guidance for reporting health equity in observational studies, globally and in studies focused on Indigenous People
  1.2. describing analysis and reporting of health equity in observational studies, globally and in studies focused on Indigenous People
  1.3. exploring and mapping diverse viewpoints of knowledge users and researchers, globally and in studies focused on Indigenous People
2. To seek wide international feedback on items to improve reporting of health equity
3. To establish consensus amongst knowledge users and researchers on reporting items for the global guidance and for the guidance focused on Indigenous Peoples in Australia, Canada, New Zealand, and the USA
4. To evaluate outcomes of collaboration between and across global guidance and guidance focused on Indigenous populations
5. To widely disseminate and seek endorsement from relevant knowledge users

## Methods

### Study design

**Fig. 1:**
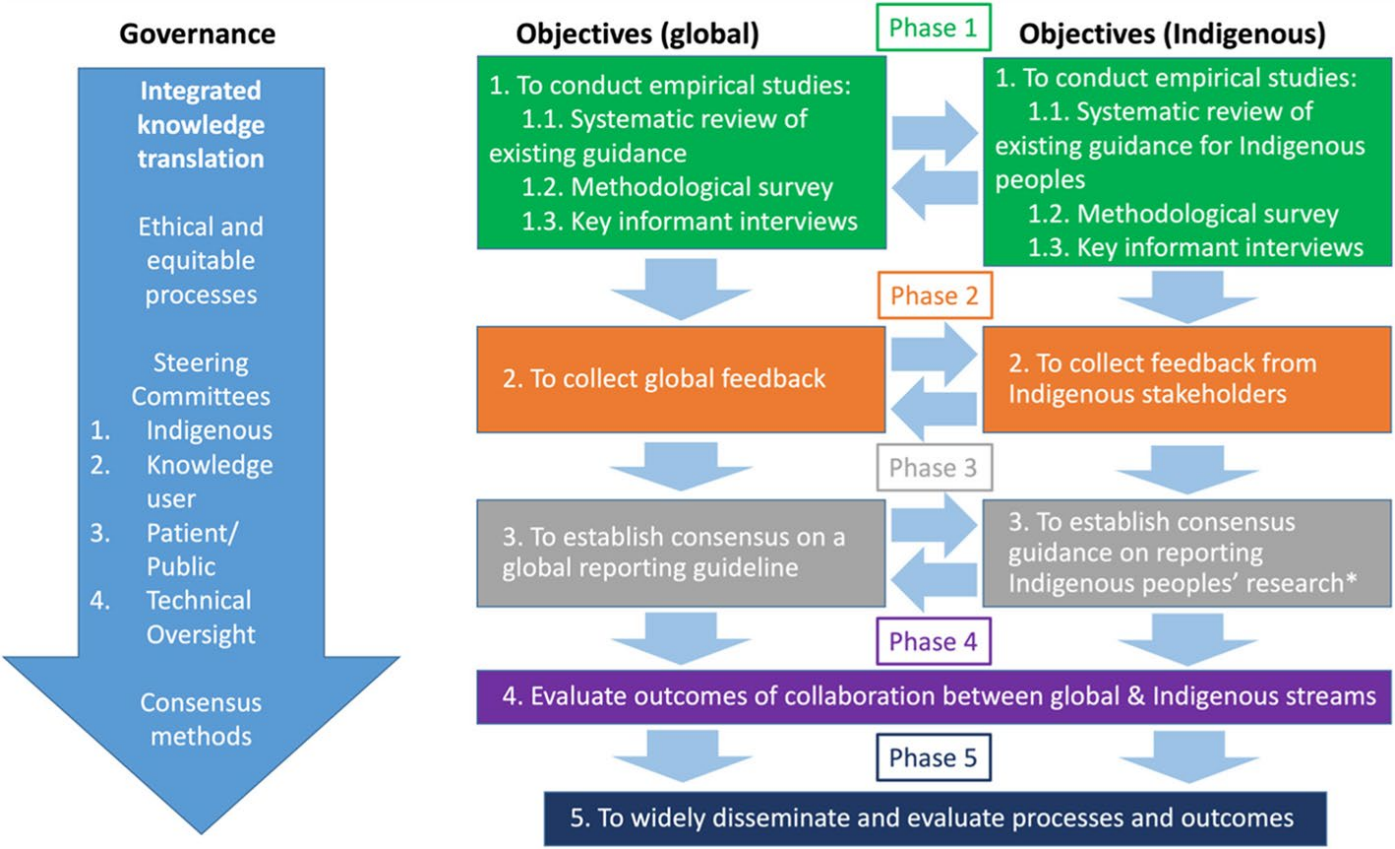
Project Overview- reporting of health equity in observational studies. Indigenous refers to Indigenous peoples living in Australia, Canada, New Zealand, and the USA

The study will comprise five phases, adapted from guidance for developing reporting guidelines by Moher et al. [60], including establishing the need for guidance, seeking comprehensive feedback, establishing consensus, evaluating outcomes of learning across streams and implementation over the span of 4 years. Members of our team have adopted this approach for other reporting guidelines on equity in systematic reviews [61, 62] and randomized trials [CONSORT-Equity] [63]. The five phases of the current study are: (1) Assess available guidance, viewpoints, and reporting of health equity in observational studies, (2) Seek wide international input using a survey on potential items to improve reporting of health equity, (3) Establish consensus amongst knowledge users and researchers on the health equity reporting guideline, (4) Evaluate the relevance of the reporting guideline for Indigenous research, and, (5) Widely disseminate and encourage uptake of STROBE-Equity (Fig. 1). In our study, we focus on Indigenous Peoples in Australia, Canada, New Zealand and the USA given the similarity in colonization experience and representation on the Indigenous Steering Committee for this project, as described above.

We will review the candidate items supported from scoping review and the items found to be poorly reported in the methods study. Study design details regarding the methods studies will be published on open access platforms or published in peer review journals when possible [64–66]. We will analyze focus groups thematically and use themes to generate additional candidate items. All the items will be complied and synthesized to develop a final list of items to be presented in an online survey. The survey will be used to gather international feedback on the suggested list of items and identify gaps where new items might be required. The results of the survey will be analysed quantitively by calculating the frequencies and percentages of each response option for each question. We will assess the agreement on the candidate items and hold the consensus meeting to discuss the final list of items. Subsequently, we will develop a writing team to wordcraft and refine the items in response to feedback on drafts, and compile examples identified by the empirical studies and expertise of the team.

**Fig. 2:**
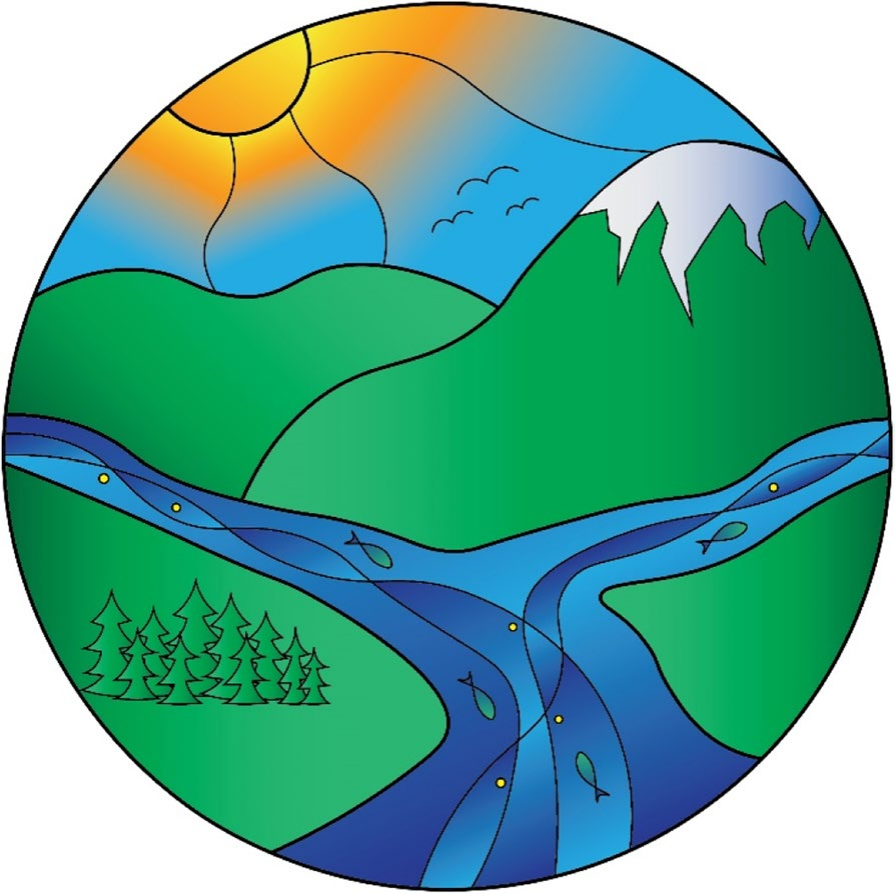
STROBE-Equity logo showing parallel global and Indigenous streams, with interconnections between them. Footnote: This figure was designed in collaboration with Claire Brascoupé (Algonquin Anishinaabe artist). The geography and landscape reflect the focus of the Indigenous stream on research with Indigenous people in Australia, Canada, New Zealand and the USA. The global stream is intended to be global and phase 2 and phase 3 of the project will seek participation and viewpoints from across the globe

We will conduct this work in parallel to the stream focused on Indigenous research led by our Indigenous principal investigator (SF), with engagement of relevant Indigenous knowledge holders and knowledge users and will follow the same steps. The two streams of research will be interconnected and empowering with opportunities for joint learning through regular communication. To articulate the connections between the two streams of research, we consulted with an Indigenous artist Claire Brascoupé (Algonquin Anishinaabe artist) to design a figure of our shared vision for parallel streams, with interconnected elements (Fig. 2). The process of co-designing this figure with our steering committees helped to establish our methods for enabling separate streams with multiple points for connection and learning from each other.

### Phase 1: assess available guidance, viewpoints, and reporting of health equity in observational studies

We will conduct six linked empirical studies on how health equity is reported in observational studies; three focused on global research and three on research on Indigenous people. We define observational studies according to those relevant to the STROBE reporting guideline as case-control, cohort, and cross-sectional studies, which collect and report quantitative data, e.g., studies conducted using administrative data, natural experiments, and prospective cohorts.

We will use an organizing framework to document evidence about health inequities defined by the acronym: PROGRESS-Plus, which stands for Place of residence (urban/rural), Race/ethnicity/culture/language, Occupation, Gender or sex, Religion, Education, Socioeconomic status and Social capital [11]. The “Plus” characteristics are other characteristics associated with health inequities such as age, disability, sexual orientation, and temporary exposure to vulnerable circumstances such as discharge from hospital and food security [67]. We will apply this PROGRESS-Plus lens to our global studies as well as to our studies focused on observational studies with, for and about Indigenous peoples.

#### Studies focused on observational studies specific to indigenous people

For each of the studies below we will conduct a parallel project focused on research with Indigenous people. We define Indigenous Peoples as “… distinct social and cultural groups that share collective ancestral ties to the lands and natural resources where they live, occupy or from which they have been displaced.” [68] Throughout the conduct of the Indigenous studies and design of the Indigenous reporting guideline, we focus on research with Indigenous Peoples in Australia, Canada, New Zealand and the USA given their similarity in colonization experiences. When engaging stakeholders in the development of the reporting guideline, we will involve representatives from these four countries. As part of the implementation and dissemination strategy, we will work towards extending the STROBE-Equity guideline to Indigenous populations in other geographies in the future. We will ensure representation and leadership from such populations accordingly. We decided that there is a need for leadership by Indigenous and allied scholars and representatives, people with lived experience, and decision-makers and also data sovereignty in interpreting results for the Indigenous reporting guideline. Designing these as two separate guideline processes allows for divergence of methods to hold true to Indigenous ways of knowing and respect importance of different types of knowledge and experience in developing this guidance, and to explore similarities and differences. We might incorporate variations in methodology to conduct the Indigenous studies in more depth, identifying not only the extent of reporting, but how steps were carried out.

#### Scoping review of guidance on reporting equity in observational studies

Despite increasing awareness about health equity by journals and funding agencies, there is no synthesis of available guidance for reporting equity in observational studies. We will conduct a scoping review of guidance in journal policies, funding agencies, and institutional ethical guidance related to criteria for inclusion, recruitment, retention, analysis, reporting and dissemination of research evidence from observational studies relevant to people who experience inequity across one or more PROGRESS-Plus factors to identify guidance supporting or refuting our candidate list of items. Scoping reviews can help clarify key concepts and definitions and identify key characteristics of the assessed concepts [69]. We will follow the transparent and rigorous methods of the Joanna Briggs Institute (JBI) for scoping reviews and the Preferred Reporting Items for Systematic reviews and Meta-Analyses extension for Scoping Reviews (PRISMA-ScR) guideline for reporting scoping reviews [69]. We will also capture guidance that contributes to informing new items.

Will conduct a comprehensive search of electronic databases such as MEDLINE, LILACS (Latin American and Caribbean Centre on Health Sciences Information), Embase, CINAHL and EbscoHost. However, we anticipate much of the information on guidance to be in grey literature sources, such as institutional reports, ethics, and funder guidelines, and journal editorial policies, so we will design a tailored grey literature search strategy with an experienced librarian scientist (TR). This search will include random sample of each of the previously listed sources as well as relevant websites (e.g., Institute of Medicine, Canadian Task Force on Preventive Health Care, and World Association of Medical Editors). We will seek to balance relevant grey literature sources from high-income countries and from low- and middle-income countries. In addition, we will ask for suggestions from our Technical Oversight and steering committees. After selecting documents, two independent reviewers will extract data on the methods of development, guidance on items related to considering PROGRESS-Plus in study design, conduct and reporting. We will synthesize the information quantitively using descriptive statistics to present the frequency of each supported or refuted candidate item [65]. We will construct tables to present the findings.

#### Methodological assessment of 320 observational studies

We will conduct a methodological assessment of reporting of health equity considerations in a random sample of 320 observational studies to establish the extent of reporting the inclusion/exclusion criteria, recruitment, retention, analysis, and interpretation of populations across socially, economically and geographically stratifying variables defined by the PROGRESS-Plus framework published in 2020 and 2021. Our team members have used such methodological assessments to inform the development of other reporting guidelines, such as for randomized cluster trials [70] and systematic reviews [71]. We chose this sample size since we expect that about half of the studies will conduct a subgroup analysis across one or more PROGRESS-Plus factors based on our prior methodological study [63]; this sample size provides 95% confidence intervals of + 6% for observed proportions of 50% reporting items of interest. This sample size has also been used in similar studies [71–73].

We will search for observational studies with help from our experienced information scientist (TR). We will stratify sampling across characteristics of interest such as focus on Indigenous People, low- and middle-income countries, and COVID-19 studies.

We will collect data on the inclusion, recruitment, retention, analysis, and interpretation of results across PROGRESS-Plus factors (sample items in Additional file 1). The data extraction form will be pre-tested on ten studies to check for clarity and feasibility of assessment and agreement between two independent reviewers. After the extraction form is finalized, two reviewers will independently collect data on whether the study and design account for PROGRESS-Plus factors in matching, adjusting for confounding, or other methods, including whether a pre-analysis plan is available. We will describe any methods used to assess subgroup effects and interactive effects or test effects in specific populations (e.g., intercept dummy variable for the group or slope dummy), ethical considerations and consideration of context (sample items in Additional file 1). Since we are aware that published manuscripts may have limited detail on health equity we will also search for study protocols of the identified studies. In addition, we will survey the authors of these studies by email to ask whether data was collected on equity variables. We will use survey strategies shown to increase response rates [74, 75]. Despite the risk of a low response rate, this survey will compare what equity variables were collected to what is reported. We will descriptively report the frequency and proportion of each candidate item reported in our sample of observational studies. We will collate the data into a table. This study will establish a baseline of existing reporting and identify potential key informants.

#### Qualitative study on key informants’ perspectives

We will conduct a qualitative study to understand stakeholders’ perceptions of how health equity is reported in observational studies, their needs regarding reporting of this information, and the barriers that may limit such reporting (e.g., privacy issues). In our prior research on health equity in randomized trials, we found that key informants identified important additional items, provided in-depth views on concepts that were not otherwise described in the literature (e.g., ethical issues), and that engaging patients/public participants was an effective way to elucidate views that were not represented in the planned survey or meeting [76].

We will conduct key informant semi-structured interviews, either individually or in focus groups, with members of the public, patients, funders, policy makers, researchers, organizations that support people who experience health inequities, knowledge users and journal editors. We will use semi-structured interviews because they are efficient and allow respondents to illustrate concepts [77]. We will design the interview guide with input from our Technical Oversight and Steering committees. An initial set of key informants will be selected from the corresponding authors of observational studies and leaders of guidance identified in the preceding steps. We will use purposeful sampling for a maximum variation of geographic diversity, types of observational studies and use of the STROBE guideline. We will expand our sample by snowball sampling, i.e., by asking each informant to identify additional possible people who might have different opinions from themselves. Theoretical saturation will determine the sample size, defined when subsequent interviews contribute no new data, based on the research question [78]. Theoretical saturation will be considered when no further new information is identified. Saturation is estimated at about 15-18 interviews, and analysis will be concurrent with data collection [78].

We will conduct a parallel and independent study with Indigenous key informants from Australia, Canada, New Zealand, and the USA including members of the public, patients, funders, policy makers, researchers, organizations that support people who experience health inequities, knowledge users and journal editors. We expect that the narrative structure of interviews applied to global interviews will apply to the Indigenous research as well. However, different issues may arise related to anti-oppressive and anti-colonial methods and Indigenous knowledge sovereignty. Thus, we will assess for saturation of concepts within Indigenous key informants. We will carry out a thematic analysis of these interviews using coding, categorizing, identifying themes and conceptualization. Two coders will verify and agree on categories and use NVivo12 software (http://www.qsrinternational.com/products_nvivo.aspx) to facilitate the qualitative analysis. This analysis will be conducted by trainees under supervision of JJ and CJP.

### Phase 2: seek wide international input on potential items to reporting of health equity

We will develop an online survey of potential items by triangulating, synthesizing and integrating findings from each of the studies conducted in Phase 1 (survey findings the scoping review, the methods review, the key informant interviews) to develop good practice examples. The steering committees and Technical Oversight will be consulted for feedback, and the draft survey will be tested with potential respondents. Our Technical Oversight and steering committees will be asked to identify at least 200 global individuals to participate in an online survey using Survey Monkey (https://www.surveymonkey.com/) on the importance of potential items and will be invited to provide open-ended comments about each item. The participants will represent different intended knowledge users, researchers who conduct observational studies, ethics board representatives, journal editors, funders, decision-makers, and patients/public. Similarly with other aspects of our project, we will seek diverse respondents to include people with experience across all aspects of PROGRESS-Plus, geographic diversity, age, gender, and country income balance. We will collect details on sociodemographic characteristics and disciplinary background. For patients/public, we will conduct face-to-face video or phone interviews according to their preference. We have used this approach successfully for other guidelines, achieving over 200 responses across diverse collaborators [63, 79]. We will use the CHERRIES checklist for reporting the survey results quantitatively [80], including tables and figures.

We will carry out a separate survey of Indigenous knowledge holders, decision-makers, and the public, and these results will be reported according to CHERRIES and summarized with descriptive statistics in tables and figures.

### Phase 3: establish consensus amongst knowledge users and researchers on health equity reporting guidelines

For the global guidance, we will hold a consensus meeting of 25-30 international, multidisciplinary and diverse collaborators to develop the STROBE-Equity extension. The Technical Oversight and steering committees will suggest people according to the following criteria: opinion leaders, standard setters, and experts in disciplines relevant to observational studies and equity (e.g., methodology, social sciences, public health, health equity), intergovernmental organizations and multilateral agencies, collaborators with diverse perspectives (e.g., funders, editors, practitioners) and patients with lived experience of health inequity. We will consider balance in participants across characteristics such as career stage/age, gender and low- and middle-income countries (LMIC)/high-income countries (HIC). At least 10% of participants will be people with lived experience, and at least 20% will be Indigenous. The number of participants is based on our experience of similar meetings as the maximum to allow meaningful discussion and is similar to the number recommended by other organizations such as the James Lind Alliance [81]. Two weeks prior to the meeting, participants will be given pre-reading materials, including results of empirical studies. To ensure sufficient time, we will hold a pre-meeting vote by all invited participants to identify areas where more discussion is needed. We will summarize evidence from the above studies (conducted in Phase 1) and exemplars of best practices for each item. We have found this promotes discussion. For each item, we will seek consensus using the Nominal Group Technique [82]. We will discuss and decide whether to publish a single manuscript on the STROBE-Equity extension or develop a short Statement paper, and a separate Elaboration paper with examples, e.g., Preferred Reporting Items for Systematic reviews and Meta-Analyses extension for Equity (PRISMA-Equity) was published as two papers [61, 62]. A writing team will be formed to develop the manuscript(s). These will be refined through iterative discussion with all participants, steering committees, and Technical Oversight members by email, face-to-face meetings, and videoconference calls. Discussion will be documented regarding the rationale for each item.

We will conduct a separate consensus meeting for guidance on reporting health equity in Indigenous research in Australia, Canada, New Zealand and USA. This meeting will be led by Indigenous peoples and will include Indigenous researchers, knowledge holders such as community elders, decision-makers, and patients/public from Australia, Canada, New Zealand and USA. The participants will be determined in collaboration with our Indigenous research steering committee. The approach will be based on community-based participatory research and Ownership, Control, Access and Possession (OCAP®) [83] and Collective Benefit, Authority to Control, Responsibility, Ethics (CARE) [84] principles. We approach this research from a relational perspective and will focus on communities and populations who are represented on the Indigenous steering committee.

The focus on Australia, Canada, New Zealand and the USA may limit applicability and relevance of this reporting guideline for Indigenous Peoples in different countries with different colonization experiences. However, we hope that our approach and results may be useful for adapting or adopting this guidance for other global Indigenous Peoples, with leadership from Indigenous Peoples in those countries.

### Phase 4: evaluate outcomes of collaboration between and across the global and indigenous streams

We plan to explicitly document the outcomes of collaboration between the parallel stream of the global guidance and the Indigenous stream focussed on Australia, Canada, New Zealand and USA. As indicated in Fig. 1, we plan to share methods and processes developed within each stream. We anticipate that there may be innovations developed in one stream than can inform the other. Documenting these examples may be helpful in designing future studies with parallel processes.

### Phase 5: widely disseminate and encourage uptake of STROBE-equity

After discussing with the journal editors involved, we plan to publish the STROBE-Equity extension as a pre-print to allow early access and feedback prior to publication. This will allow feedback from a wider audience. Also, it will allow us to begin the end of grant knowledge translation activities soon after the consensus meeting when the participants are actively engaged. As mentioned earlier, the lack of implementation tools has been described as a barrier to the uptake of reporting guidelines [85]. Thus, we will develop practical tools (e.g., checklists for journal editors and short podcasts or stories for the public) based on consultations with the steering committee that include journal editors, and we will promote these through our connections with journal editors.

While there is no systematic strategy to disseminate reporting guidelines, we will disseminate the products to a wide range of global and targeted audiences through publications and presentations, and dialogues and exchange activities with knowledge user groups. We have registered this planned reporting guideline with EQUATOR (http://www.equator-network.org/). We host a web page on the Campbell and Cochrane Equity Methods Group website (https://methods.cochrane.org/equity/) where we will post publications, and links to surveys, tweets, and other news. Our collaborators will also be asked to suggest ways to reach their communities, for instance, through dialogues and face-to-face sessions.

We will develop an evaluation framework to assess these activities using indicators such as LinkedIn and Twitter activity (numbers of tweets, retweets, likes, and reach), invited presentations (online or face to face), number of people reached by presentations, news articles and outlets, Facebook activity (likes, shares), LinkedIn likes and replications, citations of our articles or blogs in journal articles, or presentations.

### Governance plan

**Fig. 3:**
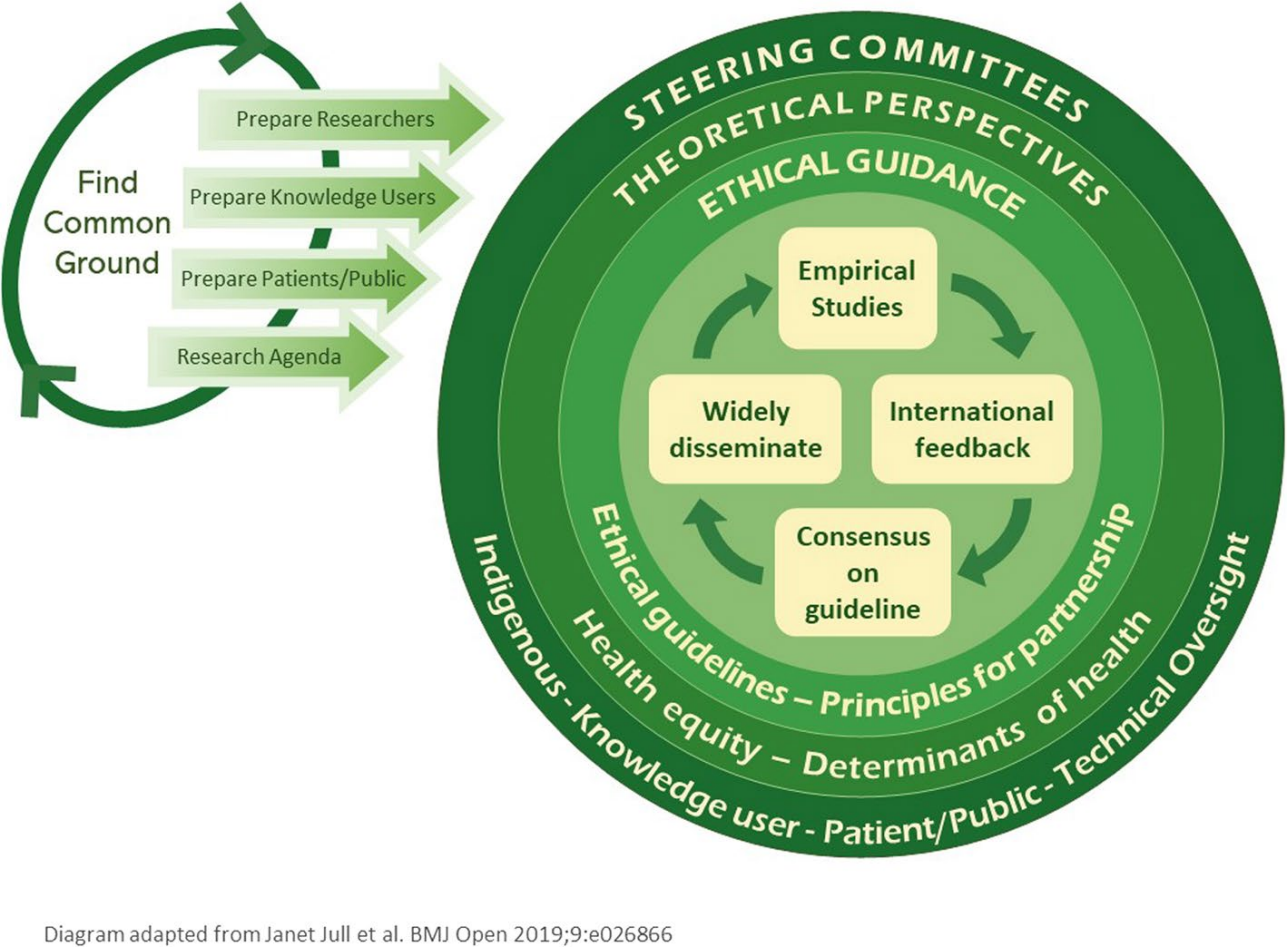
Inclusive governance plan, founded in shared goals, transparency and ethical partnerships

In developing this project, we first identified common interests in health equity in observational studies amongst our international network of patients, practitioners, and researchers with expertise in systematic reviews and scoping reviews, observational studies, health equity and knowledge translation. We established three steering committees: one for Indigenous research, one for knowledge users, and one for any other members of the public. We set up a governance structure of an executive team of four principal investigators (PIs) and a lead for each of three steering committees and a Technical Oversight committee to guide how we would work together. This team has expertise in statistics, social science, epidemiology, Indigenous health, knowledge translation, equity, reporting guidelines, lived experience of inequities, and using evidence to inform decisions. We defined terms of reference based on our prior experience, including principles of equitable partnership [86, 87]. We have co-developed an inclusive governance plan to support our plan to co-produce this research through a partnership that values different types of knowledge and participation [86, 88] (Fig. 3).

The executive team of four Principal Investigators (VW, LM, JJ, SF), a research coordinator and a post-doctoral fellow will meet regularly to consult on study methods and issues that arise during the conduct and provide quarterly updates to the whole team. The executive team will meet quarterly by video conference with the Technical Oversight Committee and the Indigenous research, patient/public and knowledge user steering committees.

Co-investigators will be invited to sign up online for all studies/papers they are interested in co-authoring. Also, we will use online tools to enable the informal exchange of ideas amongst co-investigators. Authorship for papers will be governed by terms of reference (76, 87) (Additional file 2).

Coinvestigators will participate in quarterly Technical Oversight calls for strategic direction, study design planning with their expertise in Indigenous health research. Graduate trainees will lead or co-lead empirical studies with mentorship from senior investigators.

### Patient and public engagement

We have developed a patient and public steering committee inclusive of diverse populations with an interest in health equity across dimensions of PROGRESS-Plus co-led by patient and patient representatives. The representatives have been engaged since the development of the project and will convene bimonthly to advise on the design and conduct of the studies, and the interpretation of the data. We will seek diverse viewpoints based on lived experience and values [89] and evaluate experiences of engagement using available tools [90, 91].

## Discussion

### Integrated knowledge translation

This 4-year project has been developed using an integrated knowledge translation approach with a diverse, global multidisciplinary team, including patients, authors of observational studies, statisticians, social scientists, epidemiologists, methodologists, funders, ethicists and knowledge users [86]. Our team is diverse and representative of LMIC and HIC, gender, ethnicity, career stage and disciplines. We have assembled knowledge users from (PAHO/WHO), the Royal College of Physicians and Surgeons of Canada, journal editors, the Canadian Agency for Drugs and Technologies in Health, the Public Health Agency of Canada. We will design an evaluation framework to assess the process and outcomes of this approach, including the experiences of all involved, led by JJ [87].

We will use twitter (e.g., @cochraneequity, @vawelch, @EquityStrobe), mailing lists and other fora to seek input from external collaborators. Our geographic and disciplinary diversity will help us seek diverse opinions and reach a broad audience. We will develop a one-page policy brief lay summary of each deliverable aimed at government decision-makers and journal editors. We will develop digital stories to engage the public and patients [92]. Accessibility of results will be enhanced by using reading level assessments for our policy summaries, blogs, surveys and presentations. We will translate the results into Portuguese, French and Spanish. Other translations will be encouraged (e.g., Chinese) as conducted for the CONSORT and original STROBE guidelines (https://www.equator-network.org/library/translations-of-reporting-guidelines).

### Potential challenges and mitigation strategies

A strength of our approach is the development of a parallel stream on Indigenous research which may be used as a model for creating guidance for other specific populations with different lived experience of health inequities. This requires ethical processes to facilitate culturally competent research that respects Indigenous values and ensures space for culturally safe dialogue [93]. To address this, one of our co-PIs is an Indigenous researcher with training in Ownership, Control, Access and Possession (OCAP^(R)^) [83]. Our Co-PI responsible for leading the development and evaluation of integrated KT is also trained in OCAP^(R)^ and has experience working with Indigenous communities [93, 94]. We have developed an Indigenous Steering Committee with Indigenous researchers from Australia, Canada, New Zealand and the USA to bring a high-income country and shared British colonial experience perspective. We will follow the principles of the United Nations Declaration on the Rights of Indigenous Peoples (UNDRIP) [19].

We will ensure regular communication with videoconference software, with screen share capabilities, thus minimizing environmental impact. Minutes will be distributed to allow all team members the opportunity to participate. One-on-one videoconferencing meetings (or small groups) will be used to seek specific expertise where needed.

Observational studies include diverse studies from cross-sectional surveys and natural experiments to analyses of administrative health records and longitudinal cohorts. The feasibility of taking on this broad range of studies is increased by having members of the original STROBE statement. The team includes global leaders in non-randomized studies from the Cochrane and Campbell Collaborations [95] and expertise in consensus methods [96].

A strength of our approach is the use of different methods to seek input and perspectives on the proposed items, which include an in depth key informant qualitative study, a global survey (phase 2) and a consensus process which integrates the empirical findings and survey findings with a diverse group of stakeholders. These three ways to integrate external perspectives aim to bring global perspectives to the work.

Poor implementation and lack of integration with policies, plans and the structure of research publication and knowledge management (e.g., international research registry data providers) has hindered the impact of prior STROBE guidelines [97–99]. To mitigate this risk, we have planned for 1 year of knowledge translation activities after the consensus meeting and resources for end-of-grant implementation activities, including the development, implementation, and evaluation of tools for authors, sponsors, standard setters, and editors.

### Expected impact

Given the widespread recognition of the need to redress systemic racism, sexism and all forms of discrimination in medicine [100], education [101], and justice [18], these reporting guidelines have the potential to expand awareness about the need to consider health equity in observational studies change and reporting practices on health equity in observational studies, which comprise the majority of health research. In turn, uptake and use of this guideline can substantially add to our knowledge about which health inequities are prevalent and how to redress them both within and between countries. A parallel stream focused on research with Indigenous People is a novel aspect of this guideline development, and the approach could be modelled for research with other populations who face systemic discrimination, such as people living with disabilities. We believe this will subsequently improve the evidence base on health equity in observational studies to improve social justice in policy and practice decisions.

## Supplementary Information

**Additional file 1.** Draft coding categories for methodological assessment.

**Additional file 2.** Draft Terms of reference for STROBE-Equity.

## Data Availability

Not applicable.

## Abbreviations

CONSORT: Consolidated Standards of Reporting Trials
HIC: High-income Country
JBI: Joanna Briggs Institute
KT: Knowledge Translation
LMIC: Low-middle Income Country
PAHO: Pan-American Health Organization
PROGRESS: Place of residence (urban/rural), Race/ethnicity/culture/language, Occupation, Gender or sex, Religion, Education, Socioeconomic status and Social capital
SAGER: Sex and Gender Equity in Research
PRISMA-Equity: Preferred Reporting Items for Systematic reviews and Meta-Analyses extension for Equity
PRISMA-ScR: Preferred Reporting Items for Systematic reviews and Meta-Analyses extension for Scoping Reviews
SDG: Sustainable Development Goal
STROBE: STrengthening the Reporting of OBservational studies in Epidemiology
UNDRIP: United Nations Declaration on the Rights of Indigenous Peoples
WHO: World Health Organization
