# Supplementary material for: Improving social justice in observational studies: protocol for the development of a global and Indigenous STROBE-equity reporting guideline
Source: Int J Equity Health. 2023 Mar 30;22:55. doi: 10.1186/s12939-023-01854-1 (PMC10060140; doi:10.1186/s12939-023-01854-1)
Supplement: Supplementary file 1 — Additional file 1. Draft coding categories for methodological assessment. [file 12939_2023_1854_MOESM1_ESM.docx]

# Additional File 1: Draft coding categories for methodological assessment

- - Study design (cohort, case-control, cross-sectional)
  - Funder
  - Who participated in study design (e.g. industry, academia, community, etc.)?
  - Year
  - Title/abstract
    - Are population characteristics described across one or more PROGRESS-Plus characteristics?
    - Have the limits and extents of applicability to populations across PROGRESS-Plus characteristics been described?
  - Background/rationale
    - Is health equity considered in rationale?
    - If health equity is considered, how is it defined?
    - Is a theory of change described?
    - Is context considered in the study design?
  - Setting
    - Were recruitment methods to increase participation across PROGRESS-Plus characteristics reported?
      - Describe methods
  - Participants
    - Inclusion criteria across PROGRESS-Plus characteristics
    - Exclusion criteria across PROGRESS-Plus characteristics
    - Were details of partnerships with populations and communities described?
    - For matched cohort studies – were any PROGRESS-Plus characteristics used for matching? If so how were these determined? Why were these characteristics selected?
  - Variables
    - Were outcomes selected for relevance and importance to priority populations?
  - Data sources/measurement
    - How were population characteristics obtained (e.g. age). List for each characteristic reported
  - Bias
    - Is there a possibility of bias due to some priority populations not being included?
    - Describe efforts to reduce selection bias
  - Study size
    - Were PROGRESS-plus characteristics of interest considered in determination of study size?
  - Quantitative variables
    - Were decisions related to PROGRESS-Plus analyses described?
      - Were categories defined? If so, do the authors report how these were decided?
  - Ethics
    - Was ethical approval obtained?
  - Statistical methods
    - Were PROGRESS-plus characteristics used in the design of the study to minimize confounding (e.g. matching)?
      - Which characteristics
      - How were these characteristics defined?
      - Was a rationale for their use provided?
    - Were analyses conducted to assess effect modification across PROGRESS-Plus characteristics?
      - Which characteristics
      - Which analyses and how were they carried out?
  - Results
    - Was participant flow across PROGRESS-Plus characteristics described?
      - Losses and exclusions reported across PROGRESS-Plus?
    - Were participant characteristics across PROGRESS-Plus characteristics reported?
      - Which characteristics?
    - Does the study assess whether the study sample is representative of the intended population across PROGRESS-Plus characteristics?
  - Discussion
    - Was external validity to populations across PROGRESS-Plus characteristics described?
    - Was context considered in interpreting findings?
