# Supplementary material for: Improving social justice in observational studies: protocol for the development of a global and Indigenous STROBE-equity reporting guideline
Source: Int J Equity Health. 2023 Mar 30;22:55. doi: 10.1186/s12939-023-01854-1 (PMC10060140; doi:10.1186/s12939-023-01854-1)
Supplement: Supplementary file 2 — Additional file 2. Draft Terms of reference for STROBE-Equity. [file 12939_2023_1854_MOESM2_ESM.docx]

# Additional File 2: Draft Terms of reference for STROBE-Equity

DRAFT

STROBE-Equity principles for authorship and publication strategy

This document outlines out the broad principles regarding authorship (who should be listed and in what order) for the STROBE-Equity project.

It covers written reports and papers, as well as presentations (e.g. conferences and seminars). It derives from an initial discussion at the quarterly teleconference on January 8, 2021 and subsequent discussion/revision. The principles were subsequently agreed by the team on February 4^th^, 2021.

*Principles*

1. Authorship: Not every team member should expect to be an author on every paper. Named authors need to meet criteria to qualify as authors – according to the International Committee of Medical Journal Editors - this means: 1) substantial contributions to conception and design, or acquisition of data, or analysis and interpretation of data; 2) drafting the article or revising it critically for important intellectual content; and 3) final approval of the version to be published. Authors should meet conditions 1, 2, and 3. (See <http://www.icmje.org/>). Examples of activities that could be considered “substantial contribution” include: contributing to project direction on conference calls throughout the project, identification of examples for typology or reporting items, interpretation of data, analysis of data, etc.
2. A “lead author” should expect to take the lead in drafting the paper, seeking input and comment from other authors, then revising and submitting the paper. They should subsequently also take the lead on co-ordinating the team’s response to the referee’s comments, revising the paper with help from other team members, writing the response to the journal, and submitting the revised paper.
3. The main findings papers will invite contributions from the wider research team as appropriate (assuming they meet criteria for authorship).
4. Where team members are not listed as co-authors, they will be named in the acknowledgements, on the grounds that all team members have contributed to the project through discussions at team meetings and elsewhere.
5. In the event that anyone leaves or reduces their input then their rights and contributions regarding authorship will be discussed and agreed.
6. Other papers should be agreed on a case by case basis – but as a general principle the authorship should include authors as long as they meet the ICMJE criteria.
7. An indicative list of the planned papers from the project is below. It includes the proposed lead author (and if known co-authors), and the target journal (if known). The list will be kept

updated (as details of authorship, papers etc. are likely to change over time) and will be reviewed on each quarterly call (or as needed). Team members can indicate which papers they would expect to appear on as authors, and where on the authorship list they would like to be placed.

1. No separate paper should be submitted to any journal without the agreement of the overall PIs (VW, SF, JJ, LM).
2. The above principles should also apply to conference submissions. The team will keep a record of the abstracts and conferences at which the project methods and findings are presented.

STROBE-Equity - Proposed Publications

|  | Paper | Target Journal | Lead | Co-authors (order TBD) | Status | Key Dates |
| --- | --- | --- | --- | --- | --- | --- |
| 1 | STROBE-Equity Project protocol | Implementation Science |  |  |  |  |
| 2 | Concept mapping of needs for STROBE- Equity | TBD |  |  |  |  |
| 3 | Concept mapping of STROBE and applicability to Indigenous research | TBD |  |  |  |  |
| 4 | Methods assessment of observational studies and equity reporting | Campbell Collaboration |  |  |  |  |
| 5 | Methods assessment of observational studies on Indigenous research | Campbell Collaboration |  |  |  |  |
| 6 | Systematic review of guidance about reporting equity considerations | Campbell Collaboration |  |  |  |  |
| 7 | Systematic review of guidance on inclusion and reporting related to Indigenous research | TBD |  |  |  |  |

|  | Paper | Target Journal | Lead | Co-authors (order TBD) | Status | Key Dates |
| --- | --- | --- | --- | --- | --- | --- |
| 8 | STROBE-Equity extension | Annals of Internal Medicine, TBD |  |  |  |  |
| 9 | STROBE for Indigenous research | TBD |  |  |  |  |
